# Comparison of LSTM, Transformers, and MLP-mixer neural networks for gaze based human intention prediction

**DOI:** 10.3389/fnbot.2023.1157957

**Published:** 2023-05-25

**Authors:** Julius Pettersson, Petter Falkman

**Affiliations:** Department of Electrical Engineering, Chalmers University of Technology, Gothenburg, Sweden

**Keywords:** Transformers, time series prediction, collaborative robots, human intention prediction, eye tracking

## Abstract

Collaborative robots have gained popularity in industries, providing flexibility and increased productivity for complex tasks. However, their ability to interact with humans and adapt to their behavior is still limited. Prediction of human movement intentions is one way to improve the robots adaptation. This paper investigates the performance of using Transformers and MLP-Mixer based neural networks to predict the intended human arm movement direction, based on gaze data obtained in a virtual reality environment, and compares the results to using an LSTM network. The comparison will evaluate the networks based on accuracy on several metrics, time ahead of movement completion, and execution time. It is shown in the paper that there exists several network configurations and architectures that achieve comparable accuracy scores. The best performing Transformers encoder presented in this paper achieved an accuracy of 82.74%, for predictions with high certainty, on continuous data and correctly classifies 80.06% of the movements at least once. The movements are, in 99% of the cases, correctly predicted the first time, before the hand reaches the target and more than 19% ahead of movement completion in 75% of the cases. The results shows that there are multiple ways to utilize neural networks to perform gaze based arm movement intention prediction and it is a promising step toward enabling efficient human-robot collaboration.

## 1. Introduction

Collaborative robots are becoming increasingly popular in industries (El Makrini et al., 2017). The advantages of having humans and robots in the same workspace interacting with each other are many, such as improved flexibility (Krüger et al., 2009) and increased productivity for complex tasks (Krüger et al., 2009). However, the robots are not interactive enough since they cannot interpret humans and adapt to their swift changes in behavior in a way that another human would. The main reason is that the collaborative robots today are limited in their sensory input and awareness of their surrounding environment, which makes the human responsible for avoiding collision.

Human intention prediction can be achieved using camera images and probabilistic state machines (Awais and Henrich, 2010) with the goal of determining between explicit and implicit intent. It can also be achieved using 3D-vision, speech recognition, and wearable sensors with the objective of predicting intention in hand-over tasks (Wang et al., 2021). It was proposed by Mainprice and Berenson (2013) to use a Gaussian Mixture Model and data from a Kinect camera to predict human motion, reporting about 80% classification accuracy, on 8 movement classes, after 60% of the trajectory has been observed. Other ways are to monitor eye gaze to predict an upcoming decision (Huang and Mutlu, 2016) for robot control or analyze bioelectric signals, such as electromyography, to predict human motion (Bi et al., 2019). In the paper by Haji Fathaliyan et al. (2018) it is shown that eye gaze can be used to recognize actions related to pouring and mixing a powder based drink. Shi et al. (2021) presents a way of using Earth Mover's Distance to calculate the similarity score between the hypothetical gazes at objects and the actual gazes to determine if the human visual intention is on the object or not. It was shown by Chaandar Ravichandar et al. (2016) that is is possible to use a Kinect camera to capture eye gaze and arm movements, and use that to predict the goal location of a reaching motion, reporting a success rate of above 80% after 40% of the trajectory has been observed. The work by Gomez Cubero and Rehm (2021) shows that it is possible to use an Long Short-Term Memory (LSTM)-based neural network, together with a wearable eye tracker, to predict intention regarding which object is about to be picked out of three objects in a virtual reality environment. They achieve an accuracy between 70 and 80% for test sequences that are 3–14 s long using the gaze projected on the surface where the objects are placed.

Other fields that have been rapidly expanding and could make collaborative robots smarter through an understanding of the operators behavior and intentions are: virtual reality, eye tracking, gathering and management of large datasets, and artificial intelligence.

Eye-tracking (ET) is an objective, painless, and non-invasive (Gould et al., 2001) way to gather more insight into how a person is reasoning from measurements and analysis of where the person is directing their gaze (Karatekin, 2007). It is possible to gain insight into the alternatives a person is considering or what strategy is used while performing a task, based on what a person is looking at. There are three types of interesting eye movements when observing visual attention: fixation, saccades, and smooth pursuits (Duchowski, 2017). ET has, for example, been used in an industrial context with gaze as machine control input (Jungwirth et al., 2018), to evaluate new ways to facilitate human–robot communication (Tang et al., 2019), analyze the navigational intent in humans and how they interact with autonomous forklifts (Chadalavada et al., 2020), and investigate pedestrians' understanding of an autonomous vehicle's intention to stop at a simulated road crossing (Hochman et al., 2020).

Virtual Reality (VR) can be described as a technology through which visual, audible, and haptic stimuli is able to give the user a real-world experience in a virtual environment (Dahl et al., 2017). Benefits such as being able to provide more relevant content and present it in a suitable context (Rizzo et al., 2004) are reasons to promote the use of VR. It can, for example, be used when making prototypes (Abidi et al., 2016), to train operators in assembly (Al-Ahmari et al., 2016), and improve remote maintenance (Aschenbrenner et al., 2016).

VR makes it possible to have an all-in-one system for the gathering of movement and interaction data where the developer has full control over the data and has the ability to add or remove visual and audible distractions. VR also removes the risk of injuries when the user interacts with industrial equipment in the VR environment (VRE).

Modern technologies such as ET and VR, therefore, makes it possible to collect large amounts of data, with high precision, and at a high pace (Pettersson et al., 2018). One way to process this data is through the use of an area of artificial intelligence called deep machine learning (Samek et al., 2017). The use of data and artificial intelligence has been shown to be important tools to improve industrial manufacturing (Nagorny et al., 2017; Morariu et al., 2018; Wang et al., 2018).

One particular area of machine learning that has been gaining momentum the past few years when it comes to time-series analysis is self-attention and the Transformer architecture (Vaswani et al., 2017). It has for example outperformed previous solutions for translation (Vaswani et al., 2017) and image classification (Dosovitskiy et al., 2020). The main advantages, compared to recurrent neural networks, are that the Transformers can be trained in parallel (Vaswani et al., 2017) and that they more easily learn connections between points that are far apart in time. Previously, both LSTM and Transformers have been used successfully for gaze based prediction tasks (Koochaki and Najafizadeh, 2019; Mazzeo et al., 2021; Tu et al., 2022). Another interesting architecture with similar performance to the Transformer and with less computational complexity, that does not use attention, is the “Multi-Layer Perceptron”-Mixer (MLP-Mixer) by Tolstikhin et al. (2021).

The goal of this paper is to evaluate the Transformer architecture and the MLP-Mixer as alternative solutions to intended human arm movement direction prediction instead of the LSTM network that was used in Pettersson and Falkman (2022) and compare the performance of the three approaches, with respect to accuracy for a given uncertainty threshold, time ahead of movement completion, and the execution time of a single prediction, which are defined in Sections 4–5. The data that is used to train the networks is the same as in Pettersson and Falkman (2022) to make sure that the comparison is as fair as possible. The explanations regarding the experimental setup (Section 3) and the evaluation procedure (Section 4.5) will, therefore, be the same. This is followed by a description of the current paper's two novel solutions, that are based on the Transformers and MLP-Mixer architectures, in Section 4. The prediction results and the comparison, the discussion, and the conclusions are presented in Sections 5, 6, and 7, respectively.

## 2. Preliminaries

This section provides brief descriptions of convolutional neural networks, recurrent neural networks, Transformers, “Multi-Layer Perceptron”-Mixer, and dropout as a Bayesian approximation, that are used to perform the human intention prediction.

Convolutional neural networks (CNNs) are one type of artificial neural networks (ANNs) that are more robust to shift, scale, and distortion invariance (LeCun et al., 1998) than fully connected (FC) networks, and are therefore better at detecting spatial and temporal features. It is achieved by convolving or sub-sampling the input to the layer with local receptive fields (LeCun et al., 1998) (filters) of a given size [*n* × *m*]. Each filter has *n*·*m* number of trainable weights and a trainable bias and these are shared (LeCun et al., 1998) for all outputs of the filter.

Recurrent neural networks (RNNs) are a subgroup of ANNs that are used to process sequences of data (Goodfellow et al., 2016). An RNN shares its weights across several timesteps (Goodfellow et al., 2016) whereas a FC network would have separate weights for each part of a sequence. In an RNN, the current step is not only a function of its input but also depends on all the output states previous in time (Goodfellow et al., 2016). Traditional RNNs tend to suffer from problems with exploding or vanishing error gradients (Hochreiter and Schmidhuber, 1997; Goodfellow et al., 2016) that prohibits proper learning over longer time instances. Long Short-Term Memory (LSTM) cells (Hochreiter and Schmidhuber, 1997) are designed to solve this problem using a constant error flow (Hochreiter and Schmidhuber, 1997) through the network, together with three gates that open and close in order to access it (Hochreiter and Schmidhuber, 1997). The input gate determines when the internal state of the LSTM cell is affected by the input to the cell, the forget gate handles when the cell's internal memory resets, and the output gate controls when the current state of the cell influences the error flow (Hochreiter and Schmidhuber, 1997). An LSTM network may contain multiple cells and the network learns to control each individual gate (Hochreiter and Schmidhuber, 1997) and cell.

The original Transformer by Vaswani et al. (2017) is an attention-based neural network architecture with an encoder-decoder structure, mapping one set to another, that solves natural language processing tasks. Since then, the Transformer has been adjusted in order to perform image classification with the Vision Transformer (ViT) (Dosovitskiy et al., 2020), which only uses the encoder part. This section will describe how the ViT works since that is the basis for the network used for the classifications later in this paper. The first part of the ViT splits the image into a sequence of non-overlapping patches (Dosovitskiy et al., 2020) and each patch is projected to a hidden dimension *C* that acts as the linear trainable embedding. A learnable positional encoding is then added to the embedding (Dosovitskiy et al., 2020) in order to learn the ordering of patches since self-attention inherently lacks this capability. This is then fed into the first encoder, the ViT is made up of *N*_*x*_ number of encoder blocks that are identical in size, that consists of a multi-head attention that performs self-attention in *H* parallel tracks followed by two position-wise feed forward layers separated by a non-linear activation. Self-attention is, according to Vaswani et al. (2017), a function that maps a query and a set of key-value pairs to an output, computed as a weighted sum of the values. The particular attention function used in the Transformers encoder is called Scaled Dot-Product Attention and it computes the dot products of a set of queries *Q* with all keys *K*, divide this by the square root of the dimension of the queries and keys, $\sqrt{d_{k}}$, and then apply a softmax function in order to get the weight for the values *V*, which can be summarized as follows:


(1) $$\begin{array}{l} \text{Attention}\left(Q,K,V\right)=\text{softmax}\left(\frac{QK^{T}}{\sqrt{d_{k}}}\right)V \end{array}$$

The network ends with a hidden fully connected layer and a linear classifier (Dosovitskiy et al., 2020). The ViT also utilizes skip-connections (He et al., 2016) and layer normalization (Ba et al., 2016).

The “Multi-Layer Perceptron”-Mixer (MLP-Mixer) by Tolstikhin et al. (2021) was proposed as an alternative to using CNN:s or Transformers-based architectures for image classification. Two selling points are that the Mixer network is able to achieve mostly comparable prediction results while using less memory and having less computational complexity. This gives a faster training procedure and a higher throughput (number of predictions per second) at inference. The main idea of the MLP-Mixer is to provide a simple architecture that performs two operations, mixing of features at a given spatial location and mixing between different spatial locations, in a separated way (Tolstikhin et al., 2021). These two types of mixing are present in both CNN:s and attention-based networks but in a way that is less distinct. The input to the Mixer is a sequence of non-overlapping patches that represents one image and each patch is projected to a hidden dimension *C* using the same projection matrix. The Mixer is made up of *N*_*x*_ number of Mixer-blocks that are identical in size, where each block consists of two MLP-blocks (Tolstikhin et al., 2021). The first one performs mixing between different spatial locations on the rows of the transposed input *X* and the second one mixes features at row of the input *X*. The weights of each MLP are shared for all rows and the MLPs consists of two fully-connected layers with a non-linear activation in between (Tolstikhin et al., 2021). The parameters *D*_*S*_ and *D*_*C*_ are the hidden sizes for the two MLPs respectively. The network ends with global average pooling and a linear classifier, a common way of performing classification (Tolstikhin et al., 2021). The Mixer network also utilizes skip-connections (He et al., 2016) and layer normalization (Ba et al., 2016).

Dropout is a deep machine learning method that is used to reduce overfitting (Hinton et al., 2012) by randomly ignoring, with probability *p*, each neuron in a network layer every time a training case is presented to the network. The dropout method can be used to approximate Bayesian inference (Gal and Ghahramani, 2016). It is achieved by enabling dropout at all times, not only during the training of the network, which means that the network will also randomly omit some neurons when making predictions, causing variation. The mean prediction and the model uncertainty can, according to Gal and Ghahramani (2016), be obtained by making *N* number of predictions on the same data and they suggest that *N*∈[10, 1000] should give reasonable results. This way of using dropout provides a way to reason about model uncertainty that is easy to implement and less computationally expensive than alternative methods (Gal and Ghahramani, 2016). They suggest using a probability *p*∈[0.1, 0.5] for dropping a neuron.

## 3. Experimental setup

This section will present the development of the VR environment, the test execution, the gathered data, and the way the data was processed, including selection of features and labels to train the ANN with.

### 3.1. Development of the VR test environment

The VR environment (VRE) will be visualized using the Head-Mounted Display (HMD) and the two hand-held controllers that are part of the “*Tobii Eye Tracking VR Devkit*” (Tobii AB, 2020), which is an ET solution based on the HTC-Vive. The system is capable of tracking the position and orientation of the HMD and the hand held controllers. The eye gaze is tracked with *Binocular dark pupil tracking* at a frequency of 120 Hz. This type of eye-tracking is achieved by illuminating the eyes, off-axis compared to the cameras that are used to capture images of the reflected light as it bounces off the retina and exits the eye, causing the pupil to appear darker than the rest of the eye. The images are used to calculate a gaze direction vector based on the positional relationship between the cornea and the pupil. The ET can be performed in the entire 110° field of view of the HTC-Vive HMD (Tobii AB, 2020), with an accuracy of ~0.5° and a delay of ~10 ms from the illumination of the eye until the data is available in the Software Development Kit (SDK). The eye tracker is individually calibrated to each test participant using a 5-point calibration strategy available in the SDK. The calibration is based on that the user is instructed, visually and audibly, to focus her/his gaze on 5 pre-defined points in the VRE and that gaze data is used in the SDK to calculate a 3D-model of the eye.

The 3D components in the project are modeled in the software Blender (Blender, 2023) and implemented in a VRE using Unity, a game creation engine. Unity supports VR through Steam VR SDK and custom written scripts in C# that makes it possible to implement all the desired functionality from the SDK as well as the intended test logic.

**Figure 1:**
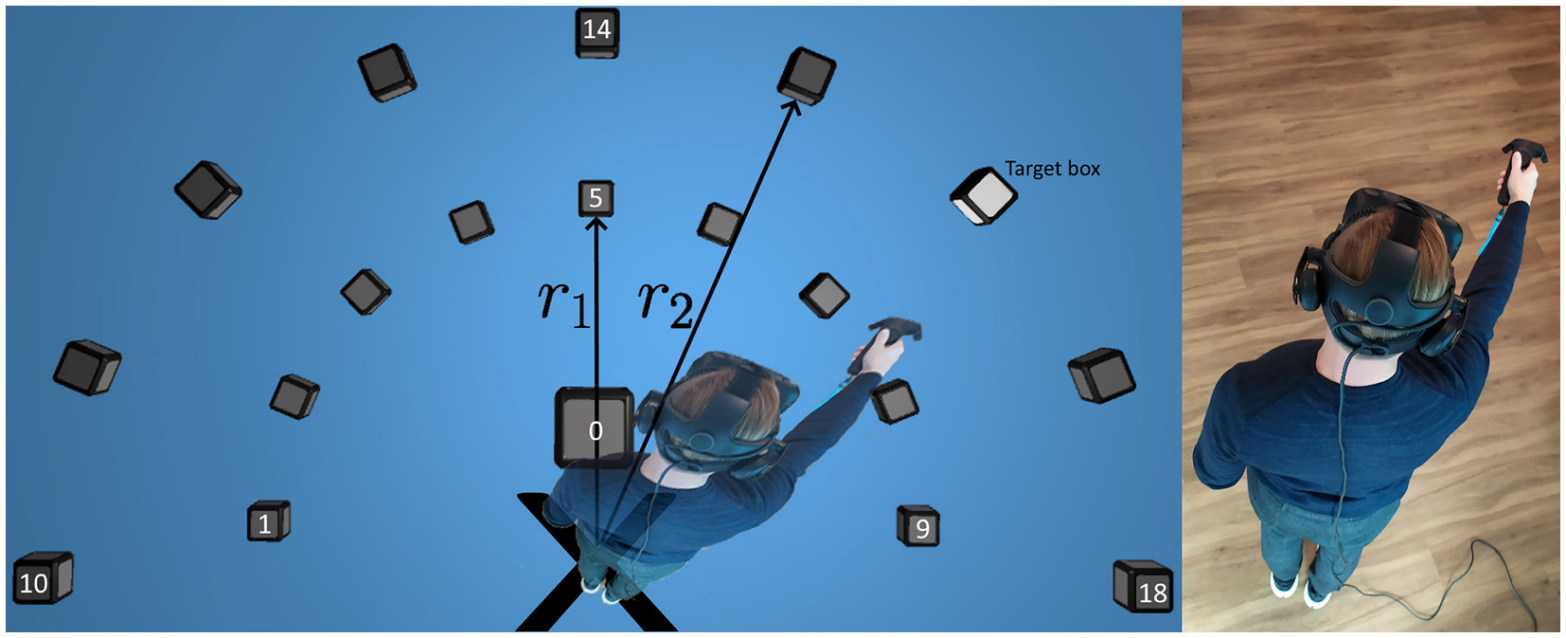
A top-down view of the block placements in the VRE, a lit target box, and the test participant moving his hand toward the target. Note that the human is not actually visible in the VRE.

The VRE designed to collect the data consists of four stages: language selection where the test participant selects whether the written instructions in the VRE should be given in Swedish or English, ET calibration, an information form where the participant enters age, gender, and whether they are right handed or not, and the last stage is the test itself. The test stage, Figure 1, is an alteration of the test in Pettersson and Falkman (2021), see below.

The test layout, shown as a top-down view in Figure 1, features an even distribution of 9 cubes each, at two different heights (*h*_1_, *h*_2_) and radii (*r*_1_, *r*_2_). One additional box, #0, is positioned 30 cm in front of the participant at height *h*_0_ and acts as a neutral position close to the body. The two arcs of cubes are generated using the fixed starting position marked by the black *X* in the figure together with individual measurements of the participant's reach based on a calibration procedure using the two controllers. The participant is instructed to raise their hands forward in three steps and click the touchpads at these locations to collect the different controller positions. The first step is to stand still with the head pointing forwards and the arms resting along the sides of the body, the second step is to raise the forearms to a horizontal level, pointing forwards, while keeping the elbows fixed against the sides of the body, and the third one is to fully extend the arms and raise them to a horizontal level, pointing forwards. The heights, *h*_1_ for the inner cubes and *h*_2_ for the outer ones, are calculated as the average distance to the floor from the controllers for the second and third position whereas *h*_0_ was set to always be located 10 cm below *h*_1_. The radii, *r*_1_ for the inner semicircle and *r*_2_ for the outer one, are defined as the average distance between the controllers and the HeadPosition. *Note that the human is not actually visible in the VRE and that the participant's only point of reference to their own body is the controller*.

Each test starts with a set of warm-up movements in random directions in order to make the participant accustomed to the VRE. The warm-up is followed by a pre-defined sequence of 76 movements using the right hand and 76 movements using the left hand. The sequence is randomized for everyone in a way that ensures balanced data and that all combinations are used. The cubes are lit up one at a time, marked as the target box in Figure 1, and the task is to reach for the box that is lit and touch it while simultaneously pressing the touchpad on the controller to make the cube disappear. After a cube has disappeared, the next cube in the pre-defined sequence is lit up after 0.2 s. The delay is used as a way to force a slower pace throughout the test and data is collected during this time. The alterations are motivated by:

- The previous test by Pettersson and Falkman (2021) was considered too long and strenuous by the participants. The current test was, therefore, halved in length, i.e., fewer number of movements in total.
- The test sequence was randomized as suggested in future improvements by Pettersson and Falkman (2021).
- Data is now collected during the short delays between the cubes appearing, this is necessary in order to be able to use and evaluate the developed network in a continuous manner that imitates a real-world system where perfect segments of data rarely are available.
- The sweeping motions across several lit boxes in a single movement were excluded due to the fact that the eye-hand movement connection is different from the search and click behavior that the randomly lit single cubes induce.

The test is launched when the test participant presses the start button in the environment. Data is then collected, in the same manner as in Pettersson and Falkman (2020) and Pettersson and Falkman (2021), i.e., the data between two pressed cubes is saved as one data point, and using the same parameters (Table 1). The data that is collected from each test participant, each test, and at each timestamp, shown in Table 1, are: the eye gaze direction vector for each eye (EyeDirection), the coordinate in the virtual room where the gaze hits (EyeHitpoint), which object is gazed upon (EyeHitObject) as well as the size and position of the pupils (PupilDiameter, Pupilposition). The head specific data that is collected are the position (HeadPosition) and rotation (HeadRotation), and the same data is also obtained from the two controllers that are held one in each hand (ControllerPosition, ControllerRotation). The general information about the user includes an anonymous participant ID, age, gender, language used, whether the person is right handed or not, as well as the date and time when the data was gathered.

**Table 1:** Table of data parameters collected during the test.

**Type**	**Parameter**
Participant	ID
Participant	Age
Participant	Gender
Participant	Date and time
Participant	LanguageIsEnglish
Participant	IsRightHanded
Test specific	BoxClicked
Test specific	Timestamp
ET	EyeDirection [*x, y, z*], (left/right eye)
ET	EyeHitpoint [*x, y, z*], (left/right eye)
ET	EyeHitObject (left/right eye)
ET	PupilPosition (left/right eye)
ET	PupilDiameter (left/right eye)
HMD	HeadPosition [*x, y, z*]
HMD	HeadRotation [θ_*x*_, θ_*y*_, θ_*z*_]
Controllers	ControllerPosition [*x, y, z*], (left/right controller)
Controllers	ControllerRotation [θ_*x*_, θ_*y*_, θ_*z*_], (left/right controller)

### 3.2. Description of the test execution

The data was collected in the VR-laboratory at Chalmers University of Technology in Gothenburg. All test participants were given the same instructions regarding putting on the headset, calibrating the eye-tracker, entering the required information, performing the height and reach calibration, starting the test, and the test specific instructions. The full set of instructions are as follows:

**Calibration Instructions:**

1. Put on the headset and adjust it such that the displays are centered in front of the eyes.
2. Receive a controller in each hand. The controllers are used to navigate the menus (using the laser pointer), touch the cubes during the test, and acknowledge all actions using the click function of the touchpad.
3. Now it is time to:

a. Choose the desired language, either Swedish or English by clicking the corresponding flag using the laser pointer.
b. Calibrate the eye-tracker:

(1) Stand still with your head pointing forwards.
(2) Press “Calibrate” using the laser pointer.
(3) Focus your gaze on the red dots that appear on the screen until they disappear, without moving your head.
(4) Press “Done” when the calibration is complete.

c. Enter the required information using the laser pointer.
d. Calibrate the height and reach parameters:

(1) Stand still with the head pointing forwards and the arms resting along the side of the body, then click the touchpad on the right controller.
(2) Raise the right forearm to a horizontal level, pointing forwards, while keeping the elbow fixed against the side of the body then click the touchpad on the right controller.
(3) Extend the right arm fully and raise it to a horizontal level, pointing forwards, then click the touchpad on the right controller.
(4) Repeat steps 3.d.i-3.d.iii for the left arm.
(5) Press “Done” when the calibration is complete or “Re-calibrate” to start over if something went wrong.

**Test Instructions:**

1. Press the “Start”-button on the screen by reaching toward it and touching it.
2. Reach for the cube that is lit up and touch it, press the touchpad while doing so to acknowledge the completion of the movement.
3. Wait for the next cube to light up.
4. Repeat steps 2 & 3 until the cubes stop emitting light.
5. Press the “Done”-button and remove the headset.

### 3.3. The obtained dataset

The dataset consists of 3,192 data points obtained from 21 participants, collected at Chalmers University of Technology. The data consists of a majority of younger adults with a higher level of education. The gender distribution of the collected data is 24% female, 76% male, and 0% other. The variations in age ranged from the youngest participant being 23 and the oldest 30 years old with an average age of 26.

### 3.4. Filtering of the data

**Figure 2:**
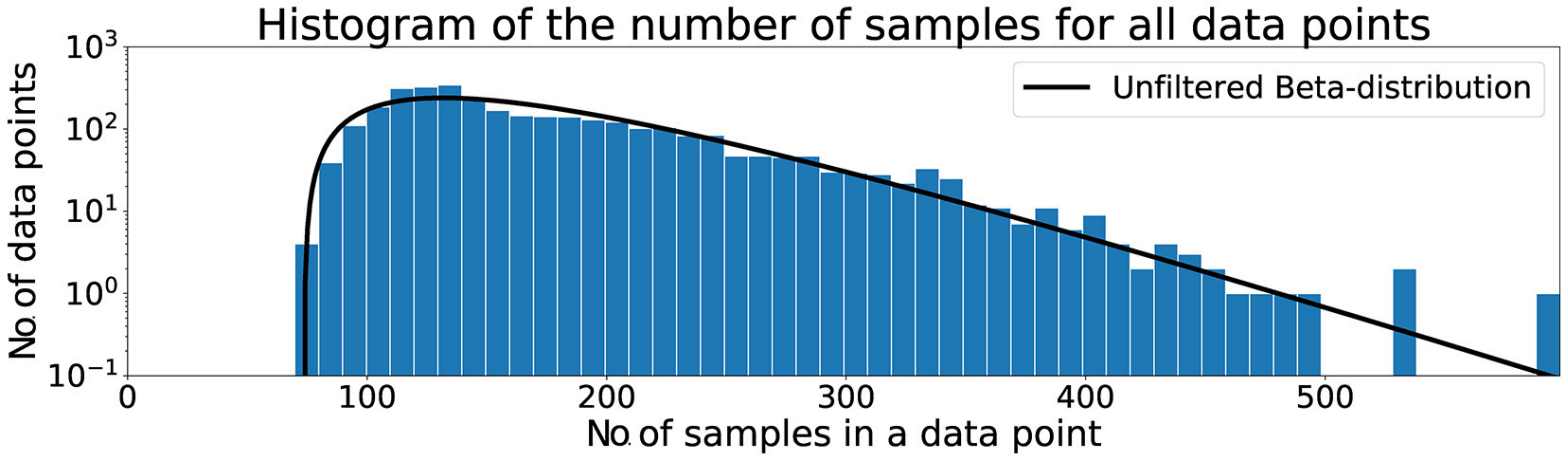
Histogram of the number of samples for all data points.

The same procedure for filtering as in Pettersson and Falkman (2020) was applied in this paper and starts with the data from the tests being loaded into the computer memory from previous storage in files on the harddrive. The warm-up sequences, described in Section 3.1, were discarded before the visual inspection of the histogram in Figure 2 that shows the length of all data points, i.e., the number of data samples that were collected from the time a box was lit until it was touched. It can be seen that the dataset contains a few outliers and the shape of the data in the histogram can be approximated using a Beta distribution, indicated by the black solid line in the figure. A threshold was set to the mean (μ) plus three standard deviations (σ) of this distribution with the values from Table 2, such that a maximum of μ + 3σ = 175 + 3*66 = 373 data samples was allowed for the data point to be used in the classification.

**Table 2:** Distribution information for unfiltered and filtered data.

**Type**	**Mean**	**Median**	**σ**	**Min**	**Max**	***N***
Unfiltered	175	152	66	74	598	3192
β-filtered	171	151	62	74	371	3141

All samples, within each data point, that contained NaN values were replaced with the gaze vector from the previous sample. NaN values appear when the ET fails to read the eye properly, the most common cause being due to the participant blinking.

### 3.5. Description of hand movement data

**Figure 3:**
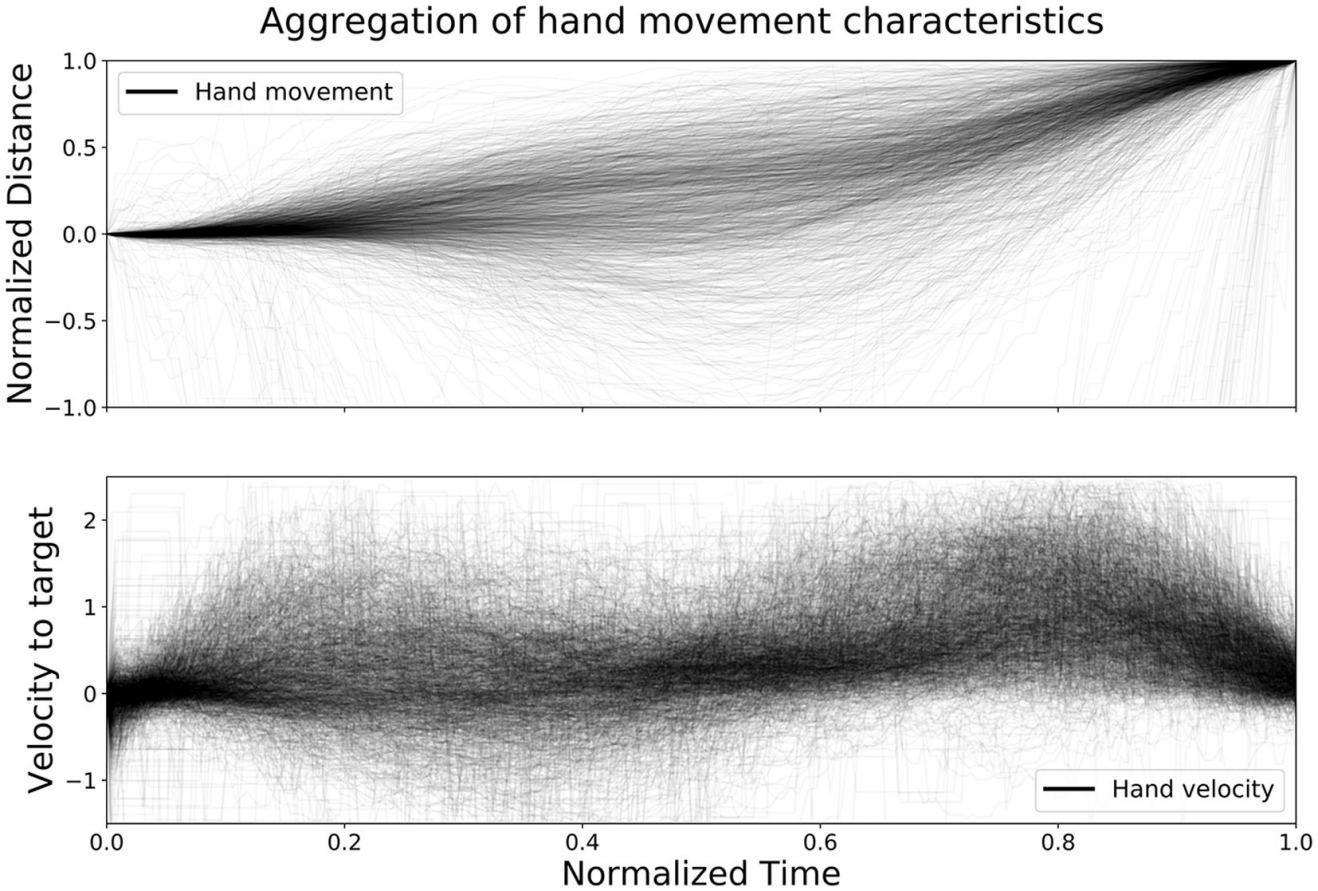
The figure shows an aggregation of all hand movements from the test set, with respect to distance left to target and velocity toward target, plotted with normalized time (Pettersson and Falkman, 2022).

Figure 3, created by Pettersson and Falkman (2022), shows an aggregation of the distance left to target and velocity toward target for all hand movements from the test set, in order to further evaluate the performance of the network with regards to time. The upper graph shows the normalized distance, *d*_*i*_, that the controller traveled from the moment the previous box was clicked until the next one, calculated at each sample *i* for each movement as:


(2) $$d_{i}=1-\frac{\left|{\text{p}}_{end}-{\text{p}}_{i}\right|}{\left|{\text{p}}_{end}-{\text{p}}_{start}\right|}$$

where **p**_*start*_ is the coordinate (*x, y, z*)^*T*^ of the controller for the first sample of the movement and **p**_*end*_ is the last one. The normalized distance was then plotted with a low opacity (alpha = 0.03∈[0, 1]) and normalized time in order to show the characteristics of all movements on the same scale. The lower graph shows the velocity, *v*_*i*_, toward the target, **p**_*end*_, at each sample *i* for each movement, calculated as:


(3) $$v_{i}=f_{s}\cdot {\left({\text{p}}_{i}-{\text{p}}_{i-1}\right)}^{T}\cdot \frac{{\text{p}}_{end}-{\text{p}}_{i}}{\left|p_{end}-{\text{p}}_{i}\right|}$$

where *f*_*s*_ is the sample frequency of the eye tracker. The velocity toward the target was then plotted with the same alpha and the same normalized time as described above. The results from the velocity calculations sometimes, due to positional tracking errors, result in unreasonable values. The velocity *v*_*i*_ was therefore removed if it exceeded 2.5 m/s. From the figure it is clear that the data is noisy and with some variation, however, a few trends are clearly emerging as well. The figure shows that there is little to no movement in the beginning of each time series followed by a segment with varying amount of movement, both toward and away from the target, up until about the halfway point. Around the halfway point the combination of the distance and the velocity graph shows a stationary segment followed by a new segment of movement that slows down toward the end. However, during the second movement segment almost all movements have positive velocity toward the target the entire time. The normalized distance data from Figure 3 was used to further investigate the time ahead of movement completion (TAMC).

### 3.6. Selection of features and labels

The features, shown in Table 3, that were used as input to the network are the combined eye gaze direction vector (*x, y, z*), obtained as an average of the separate gaze vectors from each eye, the *y*- and *z*-coordinates of the HeadPosition, and the pupil diameter, averaged between left and right eye. The *x*-coordinate of the HeadPosition was removed as it corresponds to the participant's height, which is constant during the entire duration of the test due to the fact that they remain standing and does, therefore, not provide much information to the network since the boxes are individually calibrated to the participant's height and reach. The HeadRotations were removed since the focus point of the gaze is more interesting and because of the fact that the head is often rotated in conjunction with the eyes, therefore, providing limited information to the network. The reason that information such as EyeHitPoint and EyeHitObject are not used is because they require specific knowledge of all objects in the environment, something that is possible to know in a VRE but would limit the possibility to implement the system in a real-world scenario.

**Table 3:** Description of data used in the classification.

**Type**	**Feature**
Input	AvgRLEyeDirection [*x, y, z*]
Input	AvgRLPupilDiameter
Input	HeadPosition [*y, z*]
Label	BoxClicked (0 − 9)
Label	IsUpperLevel (0, 1)

The boxes from 10 to 18 were re-labeled as 1–9 and coupled with a boolean, IsUpperLevel, that is set to one for these and zero for all others in order to fit the primary and secondary classification objectives defined by Pettersson and Falkman (2022) as:

- **Primary** - the main goal is to determine the discrete horizontal direction corresponding to the box that was clicked,
- **Secondary** - the secondary objective is to distinguish between whether the movement occurred on the upper or lower level of boxes.

The ID, age, and gender was used to manage the dataset as well as to provide some general information, these were however not used to train the network.

### 3.7. Preprocessing of the data

The selected features mentioned in the previous section, Table 3, were feature-wise normalized between [−1, 1] in order to makes sure that different value of magnitude between features does not bias the network to emphasize the importance of one feature over another. The training data was augmented by appending *N*_*T*_ = 10 nr of additional copies of the training data, without shuffling them nor adding noise, as determined by Pettersson and Falkman (2022). After augmentation, the data was transformed using a sliding window of size *w*=350and step *Ns*=70, where every window in turn was split into *Nw*=35nr of subwindows of size *ws*=10samples. The authors believe that the reason that the addition of copies works is due to the fact that we use a large step size (*Ns*=70) and that the length of the continuous sequence of training data is not evenly divisible by the window size. Hence, every copy (up to a point) will add some variety to the windowed data.

The window size, *w*=350, i.e., the number of historical gaze data samples used as network input, was set to two times the mean length from Table 2 in order to have a window that most likely spans across two different movements and thereby captures at least one transition between movements. Two different subwindow sizes, *w*_*s*_=10 and *w*_*s*_=25, where investigated by Pettersson and Falkman (2022) based on the fact that the subwindow should be able to capture saccadic eye movements that last, 10–100 ms (Duchowski, 2017), i.e., 1–10 samples. The best performing network by Pettersson and Falkman (2022) used *w*_*s*_=10 and that was used in this paper too.

The amount of data and its quality is also dependent on the step size, *N*_*s*_, that is used when sweeping over the training data. This means the number of timesteps that are skipped before the next time window is picked. The value for *N*_*s*_ should be large in order to provide the network with as unique information as possible, however, in order to not accidentally skip any movements it should not exceed the shortest movement, i.e., 73 samples. It was, therefore, set to the largest even multiple of *w*=350that satisfies this criteria, namely *Ns*=70.

Every subwindow is coupled with a label corresponding to the correct box/vertical position of the last sample of the subwindow. This means that each window, *w*=350, is divided into *Nw*=35subwindows that each get their own label and that the network makes *Nw*=35separate predictions, for each *w*=350, regarding the class that the subwindow belongs to.

The data was split in order of appearance and the proportions are: 45% of the data for training, 5% for validation, and the remaining 50% was used for testing and evaluation of the network.

## 4. Neural network design

This section will present what settings were used and how the networks were trained, a description of how the uncertainty of the networks have been estimated, the two proposed solutions, and a brief summary of the LSTM network by Pettersson and Falkman (2022) that was used to compare against.

All layers, with activation, of the networks in this paper, unless otherwise stated, uses the tanh activation function apart from the final dense layers which uses a softmax activation to enable multi-class classification ($\hat{Y_{1}}$) and sigmoid activation to enable binary classification ($\hat{Y_{2}}$).

The networks have been trained using the adam optimizer with a fixed learning rate of 3 × 10^−3^, sparse categorical crossentropy as the loss function for $\hat{Y_{1}}$ and binary crossentropy as the loss function for $\hat{Y_{2}}$. The training was performed until the validation loss stopped decreasing, terminating using early stopping. Different hyperparameter configurations were evaluated using Bayesian Optimization Tuner from the Keras Tuner library with the following custom setting: max_trials=100—the maximum number of times the algorithm runs before returning the tuned parameters and beta=5.2—the balance between exploration/exploitation, larger means more exploration. In order to evaluate the three different architectures on equal terms, a maximum number of trainable parameters allowed was set to ~7k during the tuning and initial trials, which corresponds to roughly the same size as the LSTM network (Pettersson and Falkman, 2022). The variable parts of the networks, before the UE, were selected as hyperparameters since these parts are what differ from the LSTM network and the range of values were selected such that they cover a wide range of settings without largely exceeding the maximum allowed size of the network, described above. The specific hyperparameters and their respective ranges are presented along with each network description.

### 4.1. Uncertainty estimation

The last parts of all three networks are the TimeDistributed uncertainty estimation (UE) (Gal and Ghahramani, 2016) implemented according to how its done in Pettersson and Falkman (2020). TimeDistributed refers to the fact that these parts of the network are applied to each timestep of the network (in this paper to each subwindow). The UE is constructed using dense layers (yellow squares, corresponding to FC layers) and dropout layers (green diamonds, *training=True* means that it is used also when making predictions). The UE is then followed by two TimeDistributed final dense layers, one with as many neurons as there are output classes (10) that gives the output $\hat{Y_{1}}$ and one with a single neuron that gives the binary output for $\hat{Y_{2}}$. The outputs $\hat{Y_{1}}$ and $\hat{Y_{2}}$ are obtained once for each subwindow. This way of estimating the uncertainty of the network will be used for both of the two alternative solutions presented in this paper.

### 4.2. Encoder network description—Transformer

**Figure 4:**
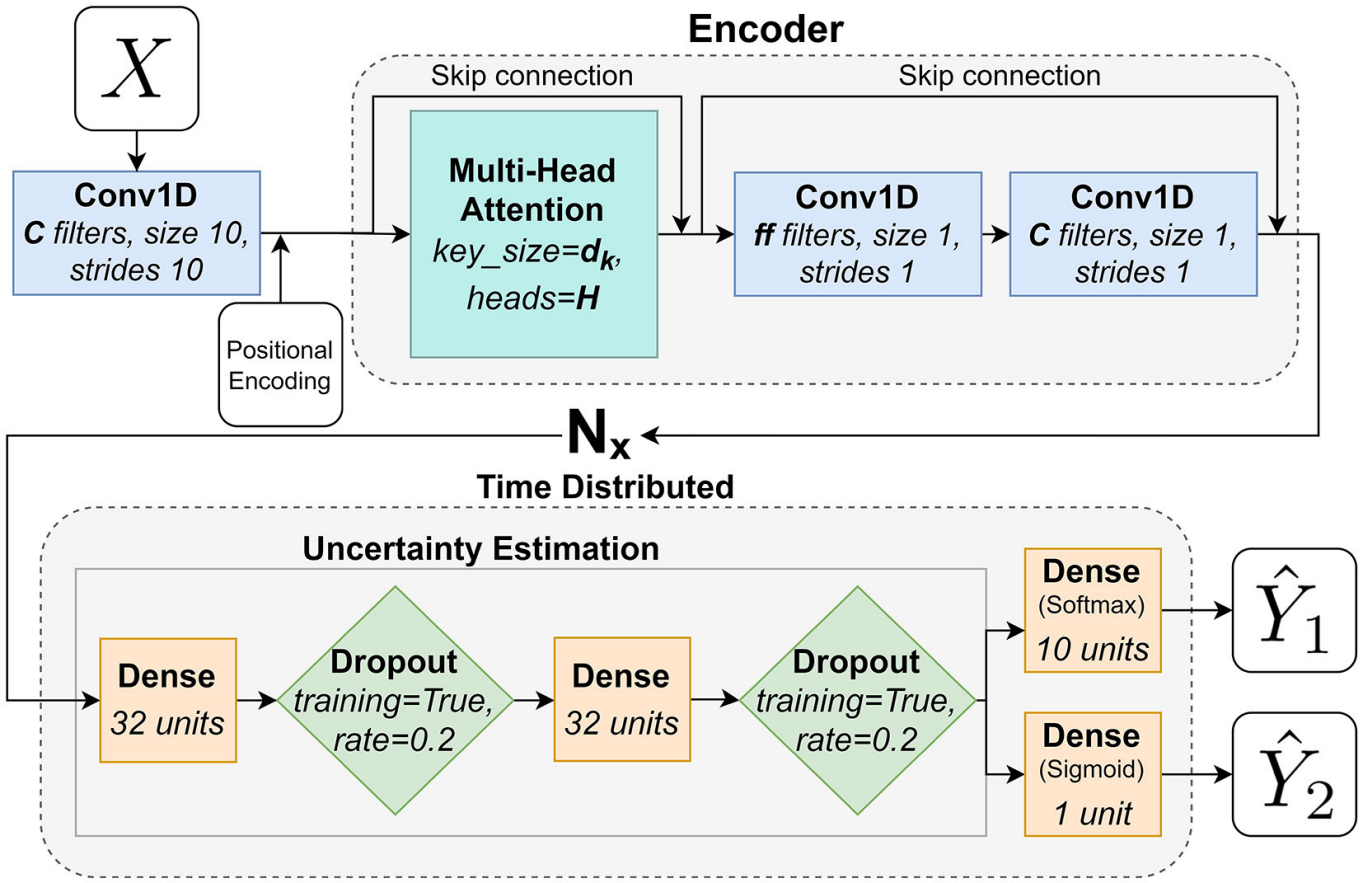
A flowchart that describes the adaptation of the Transformers Encoder architecture used in this paper.

The attention encoder, based on the ViT (Dosovitskiy et al., 2020), used in this paper can be seen in Figure 4 and works as follows: it starts with a Conv1D-layer (blue rectangles) with a fixed kernel size of 10 and a stride of 10, corresponding to the size of a subwindow (*ws*=10), and *C* nr of filters that is responsible for formatting the input data into the subwindows explained in Section 3.7. It simplifies the preprocessing, by moving the subwindowing of the data into the network, and enables the network to learn from this stage, compared to Pettersson and Falkman (2022), where the subwindows were formatted during the preprocessing. The positional encoding is a layer of trainable parameters that are responsible for learning the order of the data since that information is otherwise lost in the following attention layers. The first encoder block that starts with a multi-head attention layer (MHA, large turquoise square) that performs self-attention with key size $d_{k}=\frac{C}{H}$ in *H* parallel heads/tracks followed by two Conv1D layers with *ff* and *C* filters respectively. Both of these Conv1D-layers apply a Gaussian Error Linear Unit (GELU) (Hendrycks and Gimpel, 2016) activation. The encoder also contains two skip connections as seen in the figure. A skip connection is a summation of the output from a layer and the output from a previous layer. The encoder layer is repeated *N*_*x*_ number of times (including the first block) before the network ends with the TimeDistributed UE described in the section above and two TimeDistributed final dense layers, one with as many neurons as there are output classes (10) that gives the output $\hat{Y_{1}}$ and one with a single neuron that gives the binary output for $\hat{Y_{2}}$. The outputs $\hat{Y_{1}}$ and $\hat{Y_{2}}$ are obtained once for each subwindow. The hyperparameters, for the encoder network, that were tuned in the training phase and their respective value ranges are as follows:

- *C*∈{2, 4, 6, …24},
- *H*∈{1, 2, 3, 4},
- *ff*∈{2, 4, 6, …24},
- *N*_*x*_∈{1, 2, …, 10}.

### 4.3. Mixer network description

**Figure 5:**
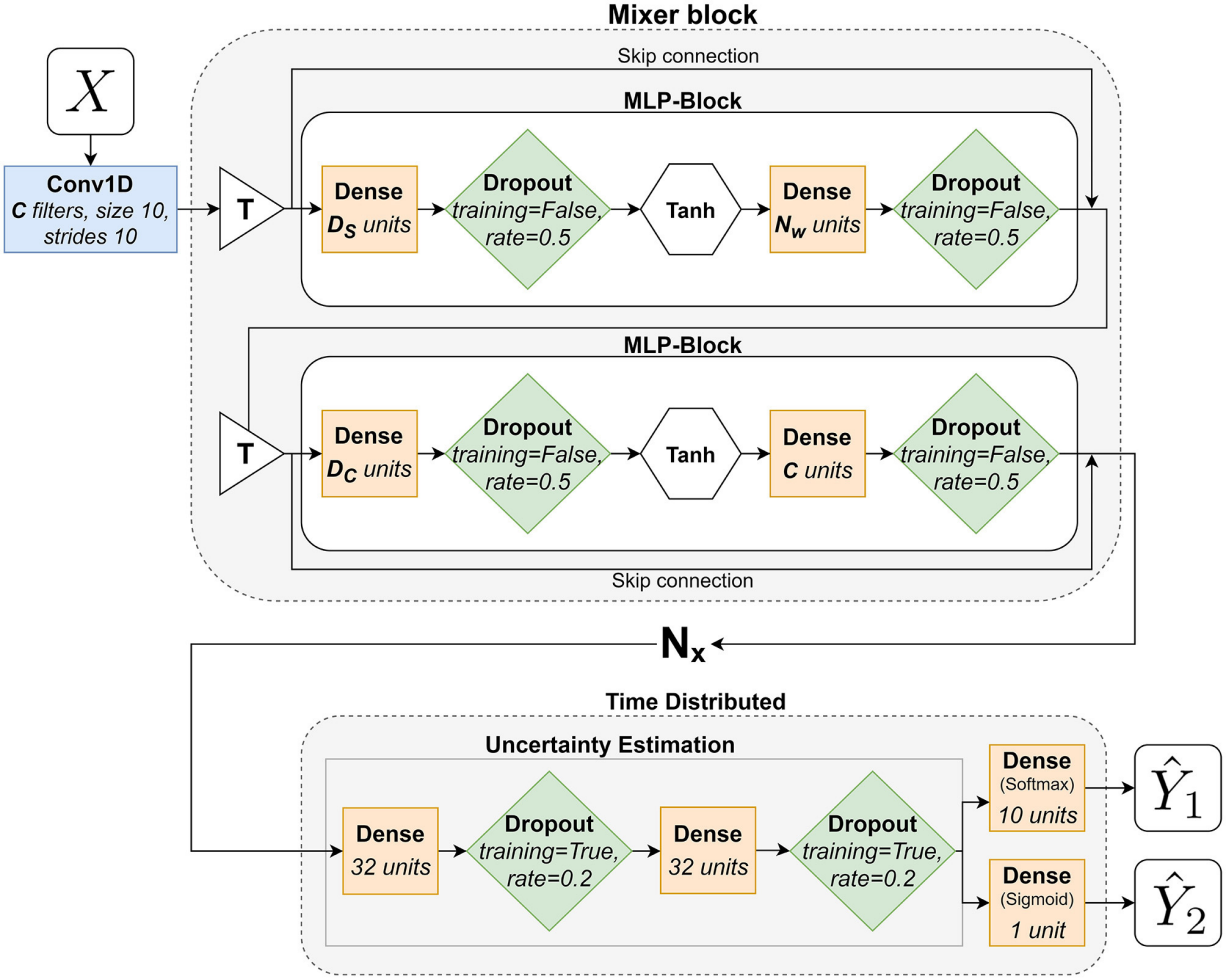
A flowchart that describes the adaptation of the MLP-Mixer architecture used in this paper.

The Mixer network, based on the MLP-Mixer by Tolstikhin et al. (2021), used in this paper can be seen in Figure 5 and works as follows: it starts with a Conv1D-layer (blue rectangles) with a fixed kernel size of 10 and a stride of 10, corresponding to the size of a subwindow (*ws*=10), and *C* nr of filters that is responsible of formatting the input data into the subwindows explained in Section 3.7. It simplifies the preprocessing, by moving the subwindowing of the data into the network, and enables the network to learn from this stage, compared to Pettersson and Falkman (2022), where the subwindows were formatted during the preprocessing. The output from this layer is fed to the first Mixer-block that consists of two MLP-blocks and two **T**-blocks (white triangles) that transposes their respective inputs. The first MLP-block performs mixing between different spatial locations on the rows of the transposed input *X* and the second one mixes features at row of the input *X*. The MLPs consists of two fully-connected layers (yellow squares), with dropout (*p* = 0.5, green diamonds), and a non-linear activation (white hexagon), tanh, in between. The parameters *D*_*S*_ and *D*_*C*_ are the hidden sizes for the two MLPs respectively. Each mixer block also features two skip connections, Figure 5, followed by a layer normalization (Ba et al., 2016) - not illustrated in the figure. A skip connection is a summation of the output from a layer and the output from a previous layer. The mixer block is repeated *N*_*x*_ number of times (including the first block) before the network ends with the TimeDistributed UE described earlier and two TimeDistributed final dense layers, one with as many neurons as there are output classes (10) that gives the output $\hat{Y_{1}}$ and one with a single neuron that gives the binary output for $\hat{Y_{2}}$. The outputs $\hat{Y_{1}}$ and $\hat{Y_{2}}$ are obtained once for each subwindow. The hyperparameters, for the mixer network, that were tuned in the training phase and their respective value ranges are as follows:

- *C*∈{2, 4, 6, …24},
- *D*_*S*_∈{2, 4, 6, …24},
- *D*_*C*_∈{2, 4, 6, …24},
- *N*_*x*_∈{1, 2, …, 10}.

### 4.4. LSTM network summary

The LSTM network by Pettersson and Falkman (2022) that will be used in the comparison can be briefly summarized as follows. The network takes the windowed and subwindowed input, feeds it to a TimeDistributed block of operations that they call ”Feature Extraction”, similar to the one found in Pettersson and Falkman (2020), that processes each subwindow separately using pooling layers and convolutions, with 4 filters per layer and a filter size of either size 1 or 3. This is then sent to the LSTM-layer, with 30 hidden units and recurrent dropout of *p* = 0.5, that learns the time dependencies of the data, and the network ends with the TimeDistributed UE explained earlier and the same two final output layers.

### 4.5. Evaluation procedure

This section provides a description of how predictions are made with uncertainty estimation (UE) (Gal and Ghahramani, 2016), what metrics that were used to evaluate the networks and how these should be interpreted, and a brief description of how to make predictions that simulates a continuous flow of data.

The difference when making a prediction with UE is that several predictions are made on the same data in order to obtain a mean value and a standard deviation of the prediction. The pseudo code for this is shown in Algorithm 1 (Pettersson and Falkman, 2020).

**Algorithm 1:** Pseudo code for predicting with UE (Pettersson and Falkman, 2020).

**Input:** X, nrOfPredictions
**Output:** Ŷ, Ŷ_*STD*_
1: predictions = []
2: **for** *i* = 0 to nrOfPredictions **do**
3: predictions[i] = network.predict(X)
4: **end for**
5: Ŷ, Ŷ_*STD*_ = mean(predictions), std(predictions)
6: **return** Ŷ, Ŷ_*STD*_

Once the means and standard deviations have been obtained from the network, these can be used to determine the network's confidence, high mean and low standard deviation, to make an accurate prediction. The implementation is shown in Algorithm 2 (Pettersson and Falkman, 2020), where a prediction is accepted if the mean minus two standard deviations is larger than a chosen threshold.

**Algorithm 2:** Pseudo code that accepts or discards a prediction (Pettersson and Falkman, 2020).

**Input:** Ŷ, Ŷ_*STD*_, lowerLimit
**Output:** Ŷ
1: **if** Ŷ−2*Ŷ_*STD*_> lowerLimit **then**
2: Accept Ŷ as the prediction for this sample.
3: **else**
4: Discard Ŷ, the network is not confident enough.
5: **end if**

The performance of the networks has been evaluated using the following custom metrics:

- *A*_*P*_ = Accuracy of predictions that are above UE threshold,
- *A*_*M*_ = Accuracy of how many movements are correctly classified at least once,
- *A*_*VP*_ = Vertical accuracy, evaluated whenever there is a box prediction.

These are more suitable to use to evaluate the network on how well it is able to utilize its notion of UE in order to predict the intended movement direction, compared to a standard accuracy metric that does not capture the aspect of UE. The reason to consider these metrics can be described as follows: *A*_*P*_ is the metric that keeps track of the accuracy of predictions that are being made, however, it is possible to achieve an accuracy of *A*_*P*_ = 100% for a very high threshold with just a single correct prediction. A result of this kind is not considered valuable since such a network would not sufficiently solve the primary objective. *A*_*M*_ on the other hand keeps track of how many movements that were correctly classified at least once. However, one way to achieve *A*_*M*_ = 100% is through a network that makes predictions all the time, without regard for the accuracy of each prediction, eventually one will through randomness be correct. This type of result is, on its own, not useful either for the same reason. Through the combination of the two metrics, *A*_*P*_ and *A*_*M*_, it is possible to evaluate how well the network is able to handle this contradictory task of being both fast to predict and correct in its prediction. *A*_*VP*_ is the accuracy score for the secondary classification objective. One way to select the threshold, *Th*_*L*_ (called lowerLimit in Algorithm 2), where a network gives the best compromise between a high accuracy and covering all movements (high *A*_*P*_ and high *A*_*M*_) is to calculate the intersection of these, referred to as combined accuracy (*A*_*I*_), on the validation set using thresholds varied between [0, 1] with a step size of 0.01.

The evaluations have been performed in a way that imitates the continuous flow of data in a real world system. This is done by making a prediction for every timestep of the test set, starting with all zeros as the input and then shifting the input data by one at a time in order to “obtain” new information. The last of the *N*_*w*_ predictions (the last subwindow) at each timestep is the one that is evaluated since that subwindow contains the most recent data.

### 4.6. Hardware and additional metrics

In addition to the metrics described above, the comparison will also include the number of parameters, *P*, that make up the networks and the execution time (*T*) of each network, defined as the time in milliseconds that it takes to perform a single prediction. All networks were trained and evaluated on the same hardware in order to ensure that the execution times are comparable. The experiments were performed on a laptop with an Intel(R) Core(TM) i7-8650U CPU and 16GB of RAM.

## 5. Results

This section will provide an overview of the training results evaluated on the validation set, the selection of networks to examine further, the performance on the test set, and the comparison against the LSTM network by Pettersson and Falkman (2022).

**Figure 6:**
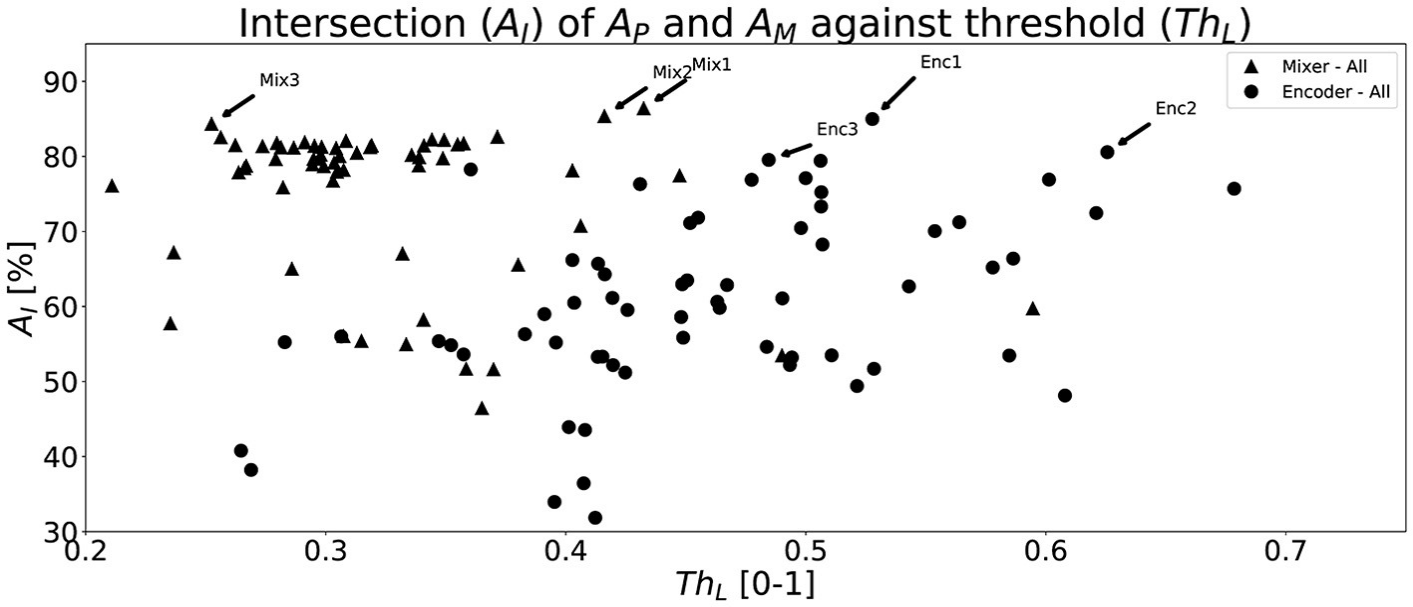
A scatter plot that shows all the intersection (*A*_*I*_) points between *A*_*P*_ and *A*_*M*_ for all network types and for each parameter configuration determined by the hyperparameter tuning described in Section 4.

The evaluation, on the validation set, of all trained networks is shown in Figure 6. The figure shows a scatter plot of all network configurations that were tested for both proposed networks. The x-axis shows the best performing threshold, *Th*_*L*_, and the y-axis the corresponding combined accuracy (*A*_*I*_), i.e., intersection between *A*_*P*_ and *A*_*M*_. It is clear that there exists many configurations that provide above 70% *A*_*I*_ whereas some fails to learn the objective, i.e., achieve low scores. The Mixer architecture seems to perform at a high level more consistently than the Encoder architecture with a large group of configurations around *A*_*I*_ = 80%. The three best network configurations, i.e., highest *A*_*I*_, of each network type from Figure 6 are shown in Table 4 along with the sizes of the networks and the thresholds, *Th*_*L*_. The Mixer architecture performs better on the validation set and utilizes a lower optimal threshold than the Encoder version.

**Table 4:** Table showing the three best configurations for each network type including their *Th*_*L*_ and *A*_*I*_ on the validation set.

**Encoder**	***C***	***d*_*k*_**	***H***	***ff***	***N*_*x*_**	***P***	***Th*_*L*_**	***A*_*I*_**
**Top 3 network configurations—Validation set**								
Enc1	16	4	4	18	2	7.02k	0.53	84.98
Enc2	12	4	3	10	3	5.79k	0.63	80.56
Enc3	12	4	3	18	2	5.25k	0.48	79.53
**Mixer**	*C*	*D*_*S*_	*D*_*C*_	*N*_*x*_	−	*P*	*Th*_*L*_	*A*_*I*_
**Top 3 network configurations—Validation set**								
Mix1	24	24	24	1		6.77k	0.43	86.41
Mix2	24	24	20	1		6.57k	0.42	85.38
Mix3	20	20	2	1		4.99k	0.25	84.33

These networks were further evaluated on the test set, at the *Th*_*L*_ obtained from the validation, and the results are shown in Table 5 along with the prediction results for *LSTM* by Pettersson and Falkman (2022). The results shows, somewhat surprisingly since the Mixers were better during validation, that the *Enc1* network is the best performing one overall with a prediction accuracy *A*_*P*_ = 82.74%, movement accuracy *A*_*M*_ = 80.06%, and vertical accuracy *A*_*V*_ = 89.10%. A good alternative to *Enc1* is *Mix3* with balanced and slightly lower accuracy scores, both of them outperformed *LSTM* in terms of accuracy. The execution time for a single prediction was measured using the python function time.perf_counter() and the measurement was repeated *n* = 10^5^ times to obtain a fair estimate. The results are presented as a mean and a standard deviation in Table 5 for each network. It is clear that the difference is negligible since the variance is larger than the difference between the fastest and the slowest network. The behavior of *Enc1* and *Mix3* will be further analyzed below, including comparisons with the behavior of *LSTM* using Figures 9, 12 from Pettersson and Falkman (2022).

**Table 5:** Table showing a performance comparison between the top performing networks from each network type, evaluated on the test set, along with the best performing LSTM network from Pettersson and Falkman (2022).

**Network**	***Th*_*L*_**	***A*_*P*_ (%)**	***A*_*M*_ (%)**	***A*_*VP*_ (%)**	***P***	***T*[*ms*]**
**Best networks from each architecture—Test set**						
Enc1	0.53	**82.74**	**80.06**	**89.10**	7.02k	31.43 ± 4.05
Enc2	0.63	79.12	65.77	87.73	5.79k	30.26 ± 3.89
Enc3	0.48	69.78	71.29	82.56	5.25k	29.96 ± 3.91
Mix1	0.43	79.09	70.51	86.90	6.77k	29.30 ± 3.89
Mix2	0.42	80.19	72.28	87.53	6.57k	29.37 ± 3.88
Mix3	0.25	76.97	77.86	87.61	4.99k	29.42 ± 3.88
LSTM	0.38	70.70	67.89	81.29	6.99k	30.39 ± 4.10

The bold values indicate the best (largest) and most interesting value for three accuracy metrics.

**Figure 7:**
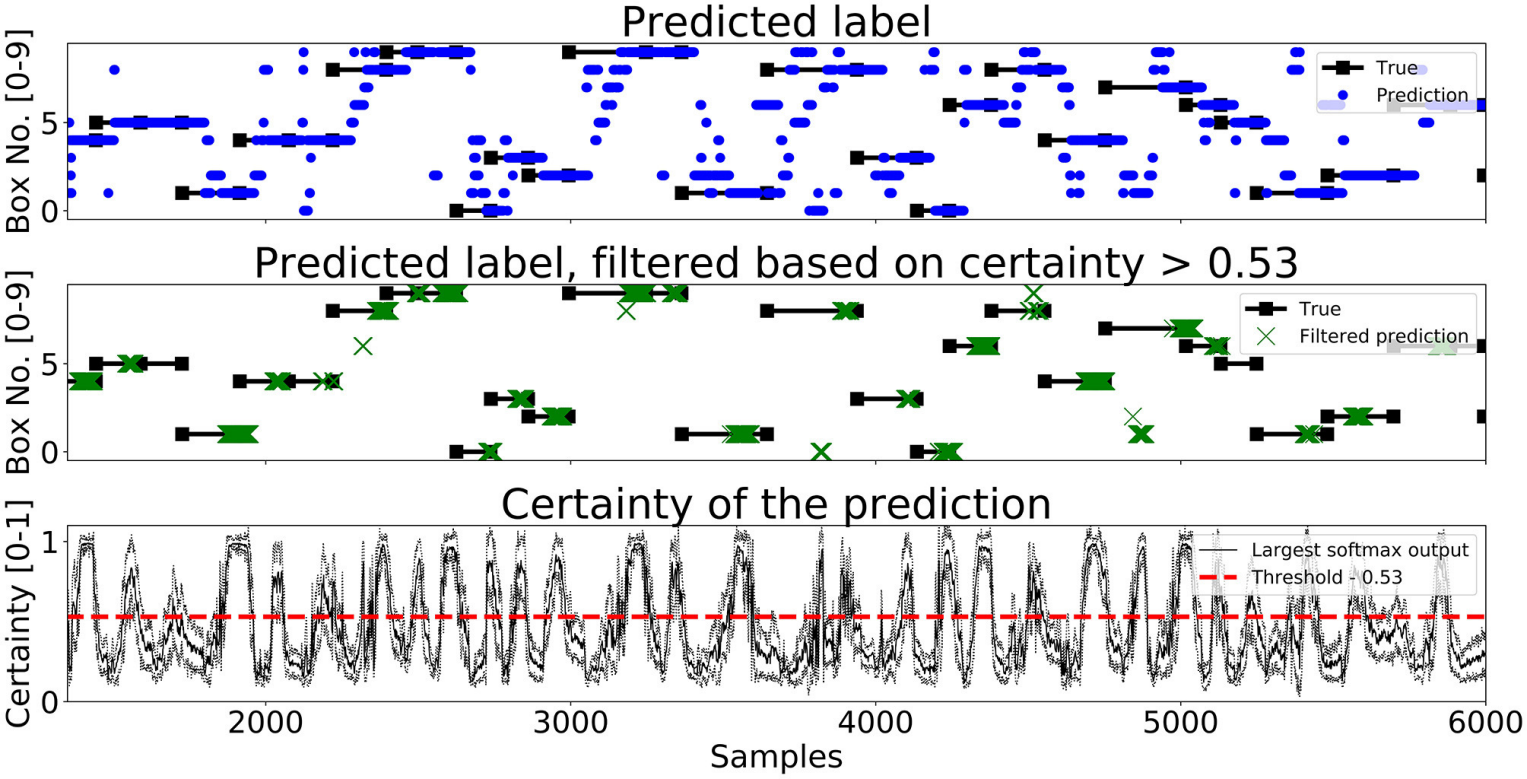
A figure that shows a prediction segment from *Enc1* obtained on the test set.

**Figure 8:**
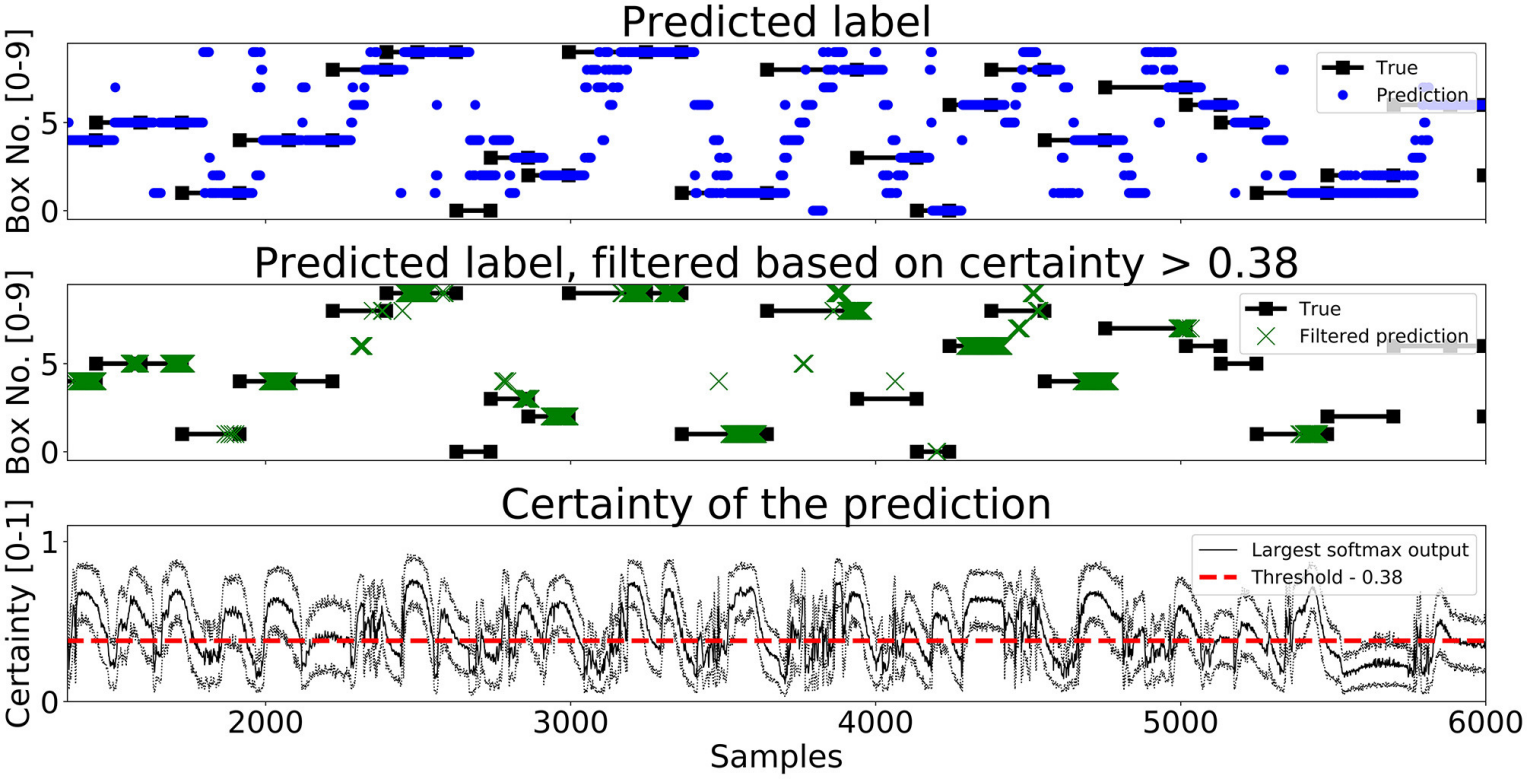
A figure that shows a prediction segment from *Mix3* obtained on the test set.

**Figure 9:**
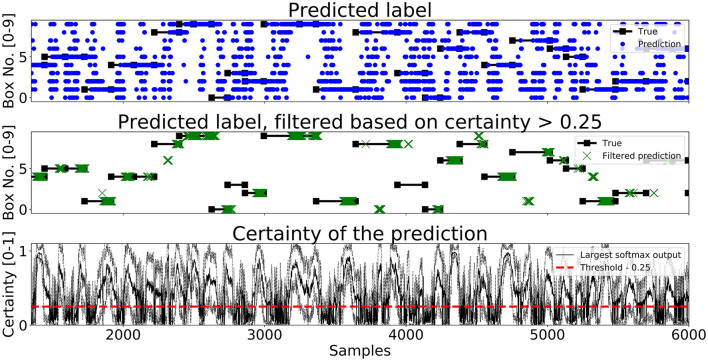
A figure that shows a prediction segment from *LSTM* (Pettersson and Falkman, 2022) obtained on the test set.

A segment of predictions on the test set for *Enc1, Mix3*, and *LSTM* are shown in Figures 7–9, respectively. The black lines with squares correspond to the true label for an entire movement, the blue dots is the unfiltered predicted label at each timestep, the green X's are the predicted labels when the certainty is above *Th*_*L*_, and finally the black line with the dotted black lines in the bottom graph corresponds to the mean softmax output plus/minus two standard deviations. It can be seen that all of the networks, after filtering on certainty, makes few mistakes and manages to correctly classify most of the movements. The bottom part of the figure displays the certainty fluctuating over time and it shows that *Enc1* often rapidly rises and falls in certainty for each movement, which indicates that the network is swift to update its certainty once it receives a new data sample. The certainty of *Mix3* fluctuates more aggressively than the other two networks and there is no clear pattern in the unfiltered predictions, however, once filtered it still predicts most movements correctly. The *LSTM* has the smoothest certainty plot but larger confidence bounds than the *Enc1*. The comparison of this segment indicates that the behavior of the certainty is not that important as long as the predictions are filtered. The fact that both the *Mix3* and the *LSTM* have larger confidence bounds is likely the reason that they have lower thresholds that gives the highest *A*_*I*_.

**Figure 10:**
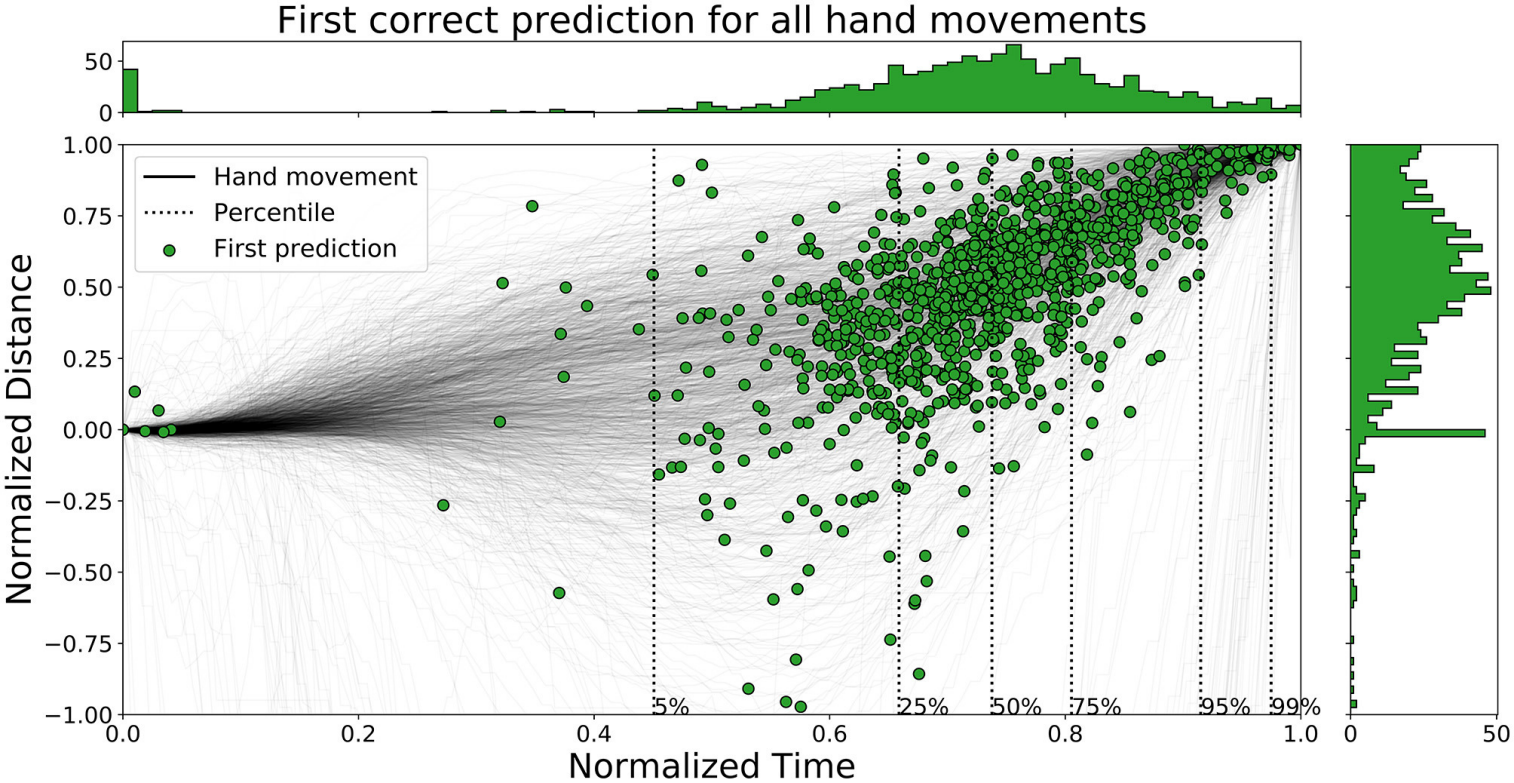
A figure that shows the first correct prediction for all hand movements from *Enc1* on the test set, plotted with normalized time and distance to target.

**Figure 11:**
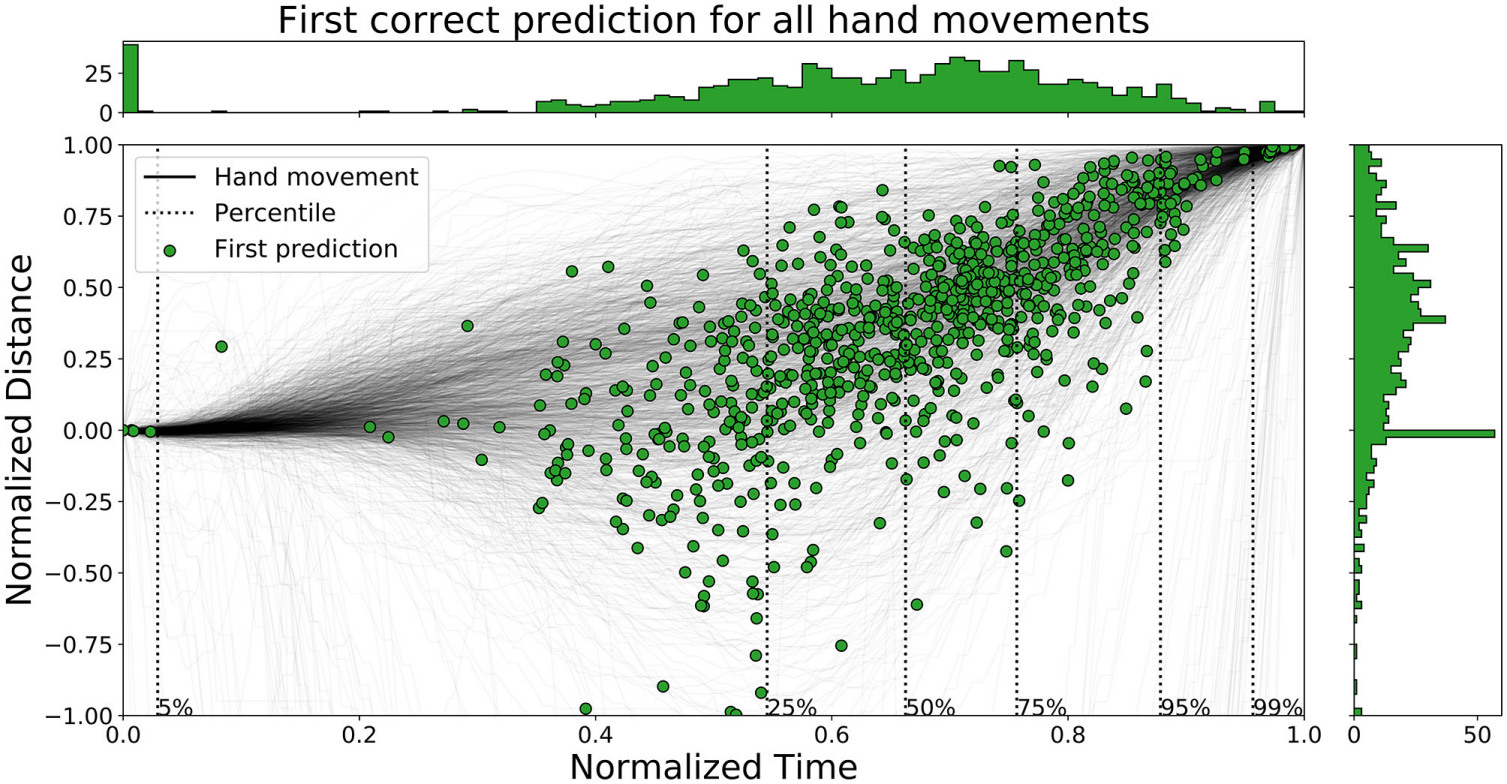
A figure that shows the first correct prediction for all hand movements from *Mix3* on the test set, plotted with normalized time and distance to target.

**Figure 12:**
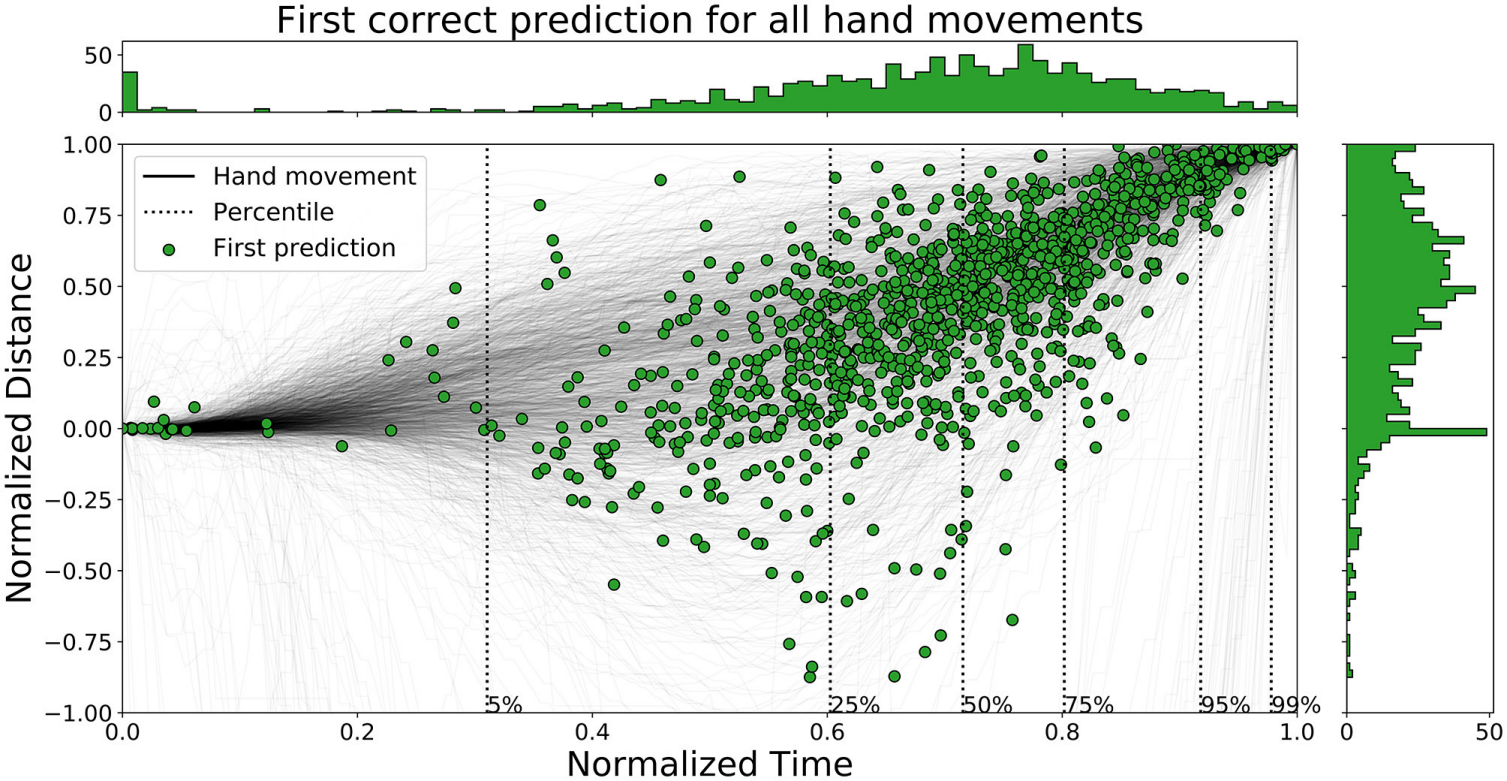
A figure that shows the first correct prediction for all hand movements from *LSTM* (Pettersson and Falkman, 2022) on the test set, plotted with normalized time and distance to target.

The time ahead of movement completion, TAMC, is analyzed for all three networks in Figures 10–12. The figures show the first correct prediction from each of the movements that were correctly classified at least once together with the hand movement data described in Section 3.5. The 5th, 25th, 50th, 75th, 95th, and 99th percentiles, with respect to time, were added to the plots and their values are summarized in Table 6. The histograms, top and right, shows the distributions of correct predictions with regards to the normalized time and normalized distance respectively.

**Table 6:** A table that summarizes the normalized time values for the percentiles of all the evaluated networks.

**Percentile**	**5**	**25**	**50**	**75**	**95**	**99**
Enc1 - Normalized time [0, 1]	0.45	0.66	0.74	0.81	0.91	0.97
Mix3 - Normalized time [0, 1]	0.31	0.6	0.72	0.8	0.92	0.98
LSTM - Normalized time [0, 1]	0.03	0.55	0.66	0.76	0.88	0.96

The distributions of *LSTM* are more spread out, for both time and distance, compared to the other two networks. The network is slightly faster than the other two since the concentration of points are shifted lower to the left. *Enc1* has the most compact distribution of points, concentrated to the upper right. This means that the network is slightly slower at detecting movements. *Mix3* looks like a combination of the other two networks since its time distribution is similar to *LSTM* and the distance distribution is more concentrated to the upper half as in *Enc1*.

Pettersson and Falkman (2022) argued that the first 5% of the correct predictions for the *LSTM* are most likely “lucky-shots”. These network predictions occur as the network sticks to the same prediction for the next movement, which due to the random box sequence sometimes was the same target twice in a row. They are called lucky since the test person, and therefore the network, can not know the next target for the first 0.2 s, i.e., first 5.4 to 27.0% of the movements for the max/min duration, due to the delay that was inserted between each task in the VRE. These “lucky-shots” are less prominent in both *Enc1* and *Mix3*.

The results in Table 6 shows that *LSTM* is the fastest at detecting movements for the lower percentiles, followed by *Mix3*, and then *Enc1*. However, the differences between the three networks decrease toward the end of the movement durations.

## 6. Discussion

This paper builds upon the work presented in Pettersson and Falkman (2022) where eye gaze and movement data was gathered, and used to train an LSTM network to perform gaze based arm movement prediction. Using the same data, this paper has provided two additional solutions to the classification objectives, presented in Section 3.6: **Primary** - *determine the discrete horizontal direction corresponding to the box that was clicked* and **Secondary** - *distinguish between whether the movement occurred on the upper or lower level of boxes*. A comparison with respect to accuracy for a given uncertainty threshold, time ahead of movement completion, and the execution time of a single prediction using the three methods is also presented. It is clear from the results on the test set that the LSTM network is outperformed by the two proposed alternative solutions in terms of accuracy. However, the search for network configurations is not exhaustive and there may therefore exist more accurate configurations of each of the three networks. The best network, *Enc1*, reached an accuracy of 82.74% for the primary objective, correctly classifies 80.06% of the movements at least once, and an accuracy of 89.10% for the secondary objective.

The validation results, somewhat surprisingly, indicated that the Mixer architecture outperformed the Encoder whereas the results by Tolstikhin et al. (2021) showed the opposite. However, this changed for the evaluation on the test set where the prediction results came out as expected, i.e., the Encoder had higher accuracy while the Mixer runs faster. The dissonance between results on the validation set and the test set may indicate that the Encoder generalizes better to new data and/or that the Mixer were somewhat over-trained toward the quite small validation set. It is also important to acknowledge that the dataset that is used in this paper is relatively small, however, with the results in mind it is deemed sufficient as a proof of concept. The fact that the use of data augmentation improves the results could be seen as an indicator that the developed network could benefit from training on a larger dataset.

It was observed, during the initial experiments with the networks, that the Encoders were highly sensitive to the learning rate (*lr*) of the optimization algorithm. A *lr* close to 10^−3^ seem to perform well whereas values approaching either 10^−2^ or 10^−4^ gave poor results.

The results in Table 6 shows that *LSTM* is the fastest at detecting movements for the lower percentiles, followed by *Mix3*, and then *Enc1*. However, the differences between the three networks decrease toward the end of the movement durations. One possible explanation to the fact that *LSTM* achieved the highest TAMC with the lowest accuracy, and that the other two networks showed decreasing TAMC with increased accuracy, may be that a network with a more aggressive prediction strategy, i.e., making predictions without necessarily being right, leads to early, correct answers more often than waiting for a high enough certainty that the prediction is correct.

One source of error is that the networks' sometimes continues to make the same prediction, without changing certainty, even though a new movement sequence has started. An explanation could be that the networks have not fully learnt how to determine when a new movement sequence starts. However, another possible explanation may be that the test person lingers with his/her gaze at the box after clicking it while waiting for the next light cue, which in turn would give the networks' no reason to believe that the target has changed.

The slower shift in certainty of the *LSTM* network, compared to the other two, may be explained by the way the three architectures handles historical data. An RNN takes the cumulative information of all previous data samples into account together with the contribution of the current timestep, whereas the attention module of *Enc1* and the mixer block of *Mix3* recalculates the importance of all historical points at each timestep, which could make them faster to adapt to new information.

The execution times presented in the Section 5 shows negligible differences between the three networks. However, more importantly it can be observed that all networks runs slower than the sampling frequency of the eye tracker (120 Hz). This means that the current solutions are not able to make predictions for every new incoming data sample in a real-time application. However, the results are still useful since the long execution times could potentially be adapted to by making predictions only after a few new data samples instead of every new gaze input. Making predictions every *n*_*p*_:th sample was suggested by Pettersson and Falkman (2022) as a way of reducing computational requirements. A suitable *n*_*p*_ for the presented solutions should be chosen based on the lower limit of the execution time in relationship with the sampling frequency.

## 7. Conclusions and future work

This paper has shown that both Transformers and MLP-Mixers are viable neural network architectures to use for the gaze based prediction of the intended human arm movement direction. It was also shown that both the proposed methods are able to outperform the previous LSTM network on all accuracy metrics. The best Transformers encoder achieved the highest accuracy for all metrics and another good alternative is the smallest Mixer network with slightly lower accuracies. All networks had similar execution times, suggesting that the choice of architecture may in the end depend on other factors related to the application. The best developed encoder network achieved an accuracy of 82.74%, for predictions with high certainty, on continuously incoming data and correctly classifies 80.06% of the movements at least once. The movements are, in 99% of the cases, correctly predicted the first time, before the hand reaches the target and more than 19% ahead of movement completion in 75% of the cases, which corresponds to about 239 ms for the median movement duration. The results shows that there are multiple ways to utilize neural networks to perform gaze based arm movement intention prediction and it is a promising step toward enabling efficient human-robot collaboration.

Future work could revolve around applying the presented methodology to new gaze based prediction objectives, investigate how the different architectures compare for longer series of gaze data, or analyze if the performance is affected by the proximity between boxes or the height placements. It could also be the implementation of a similar and more industrial task, like a kitting station, were predictions could be evaluated in real-time.

## Data availability statement

The raw data supporting the conclusions of this article will be made available by the authors, without undue reservation.

## Ethics statement

Ethical review and approval was not required for the study on human participants in accordance with the local legislation and institutional requirements. Written informed consent for participation was not required for this study in accordance with the national legislation and the institutional requirements.

## Author contributions

JP and PF contributed to conception and design of the study. JP developed the VRE, implemented the artificial neural networks, and wrote the first draft of the manuscript. All authors contributed to manuscript revision, read, and approved the submitted version.
